# Submerged goiter proven to be metastatic infiltration of a neuro-endocrine Merkel cell carcinoma

**DOI:** 10.1186/2193-1801-3-46

**Published:** 2014-01-24

**Authors:** Nikolaos Tsoukalas, Menelaos Zoulamoglou, Maria Tolia, Evangelos Bournakis, Elin Ronne, Vasileios Barbounis

**Affiliations:** Department of Medical Oncology, “401” General Military Hospital, Gennimata N. 10-12, 11524 Ampelokipi Athens, Greece; Department of Medical Oncology, “Hippocration” General Hospital, Athens, Greece; Department of Clinical Therapeutics, “Alexandra” Hospital, University of Athens School of Medicine, Athens, Greece; Department of Pathology, “Evaggelismos” General Hospital, Athens, Greece

**Keywords:** Metastasis, Neuroendocrine tumours, Thyroid, Merkel cell carcinoma, Submerged goiter

## Abstract

**Background:**

Merkel cell carcinoma (MCC) is an uncommon neuroendocrine cutaneous carcinoma. Metastases to the thyroid gland are rare and may present diagnostic difficulties.

**Case presentation:**

A 73-year-old woman presented with a hard mass in the adipose tissue of the right inguinal area. This mass was surgically excised and the histology examination showed the existence of a MCC. CT scans revealed a sizable lesion with imaging features of a submerged goiter, invasive to the upper mediastinum. The patient received chemotherapy following by locoregional radiotherapy at the bed of the excised lesion. During the next 10 months the patient was asymptomatic, serum markers values were normal and CT scans findings were stable. However, afterwards NSE and chromogranin values raised and CT scans revealed an enlargement of the submerged goiter. The patient became symptomatic, mainly experiencing respiratory inconvenience. Surgical excision of the right lobe of the thyroid gland was decided and performed without any complications. The histopathology examination showed infiltration of the thyroid gland by a neuroendocrine carcinoma with characteristics compatible with MCC.

**Conclusions:**

The rare case of metastatic infiltration of the thyroid gland by a MCC based on histological and immunohistochemical findings was described. This case report is of clinical significance indicating that by any abnormal finding in the thyroid gland in patients with a malignant disease, the diagnostic approach should always contain consideration of metastasis from the primary tumor.

## Background

Merkel Cell Carcinoma (MCC) is an uncommon neuroendocrine cutaneous carcinoma which is characterized by high incidence of early loco-regional relapse and distant metastases (Poulsen 2004).

## Case presentation

**Figure 1:**
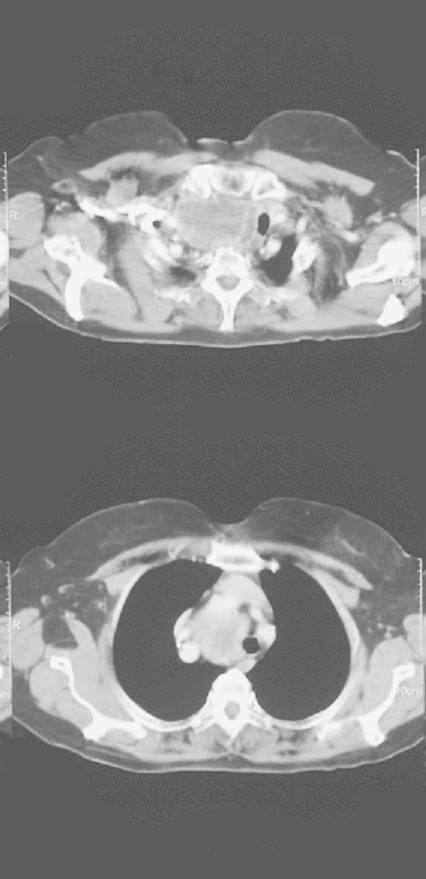
**CT scan large mass in the right lobe of thyroid grand invasive to the upper mediastinum.**

A 73-year-old woman with no prior medical history, presented with a hard mass (diameter = 46 mm) in the adipose tissue of the right inguinal area which was confirmed by CT scan (Nov 2009). A month later, this mass was surgically excised and the histology examination showed a poorly differentiated small cell carcinoma, with histopathologic features of a Merkel cell carcinoma. CT scans revealed a sizable lesion with imaging features of a submerged goiter, invasive to the upper mediastinum (diameter = 66 mm) with no enlarged mediastinal or axillary lymph nodes (Figures 1: large mass in the right lobe of thyroid grand invasive to the upper mediastinum). At the time no further diagnostic evaluation of the submerged goiter took place.

**Figure 2:**
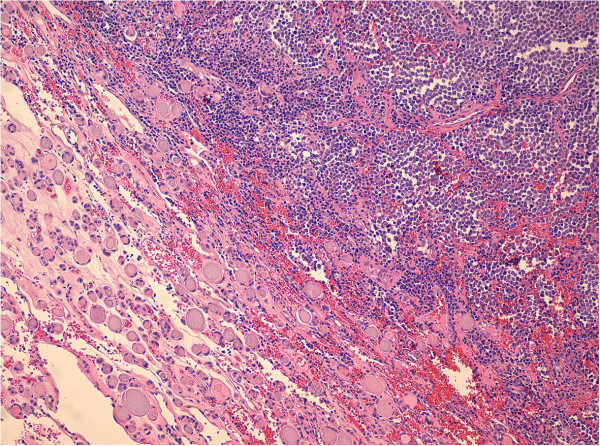
**H-E staining showing both thyroid follicles in the down left part and tumor cells in the upper right part, original magnification ×100.**

Afterwards, the patient was admitted to our department for further clinical evaluation and treatment. Serum NSE and chromogranin values were 21,8 ng/ml (< 16,3 ng/ml) and 7,6 ng/ml (< 5,6 ng/ml) respectively. Initially, the patient received chemotherapy with the regimen cisplatin 75 mg/m^2^ day1 and etoposide 100 mg/m^2^ day1-3 (6 cycles every 3 weeks) following by locoregional radiotherapy at the bed of the excised lesion (total dose 45 Gy). Both of these treatments were well tolerated. Three months later (Jan 2011) serum NSE and chromogranin values were within normal limits and the subsequent CT scans revealed stable disease. During the next 10 months the patient was asymptomatic, serum markers values were normal and CT scans findings were still stable.

However, in November 2011 follow up NSE and chromogranin values raised to 26 ng/ml and 9,2 ng/ml respectively. In addition to that, CT scans revealed an enlargement of the submerged goiter which was invasive to the upper mediastinum, displacing trachea to the left, still without enlarged mediastinal or axillary lymph nodes again. Moreover, an ultrasound of the thyroid gland confirmed a significant increase of the size of the submerged right lobe. The patient became symptomatic, mainly experiencing respiratory inconvenience. Surgical excision of the right lobe of the thyroid gland was decided and performed without any complications.

**Figure 3:**
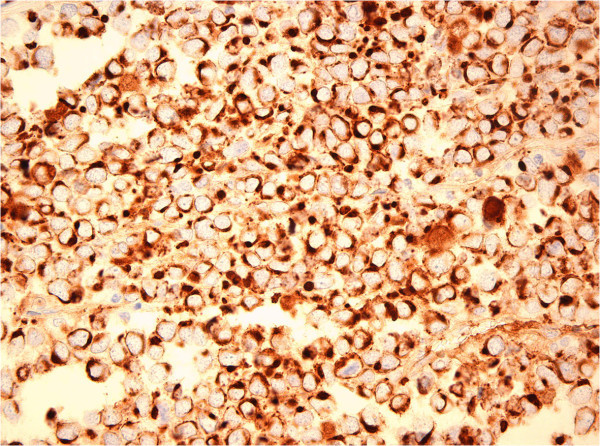
**Immunoperoxidase staining for CK20 showing positive tumor cells, original magnification ×400.**

**Figure 4:**
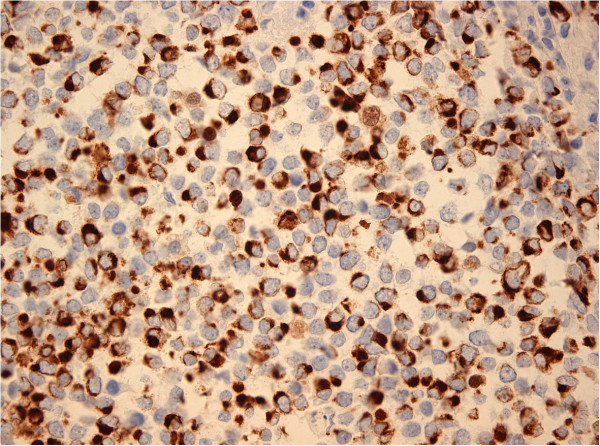
**Immunoperoxidase staining for NF showing positive tumor cells, original magnification ×400.**

The histopathology examination showed infiltration of the thyroid gland by a neuroendocrine carcinoma with characteristics compatible with Merkel cell carcinoma. The tumor consisted of uniform, small to medium sized cells with a round nucleus, finely dispersed chromatin, inconspicuous nucleoli and scant cytoplasm (Figure 2: hematoxylin-eosin-staining H-E showing both thyroid follicles in the down left part and tumor cells in the upper right part, original magnification ×100). There were numerous mitoses and areas with necrosis. The tumor was characterized of positive immunohistochemical reaction for antibodies to cytokeratin CAM 5.2, cytokeratin 20 CK20 (Figure 3: immunoperoxidase staining for CK20 showing positive tumor cells, original magnification ×400), neurofilaments NF (Figure 4: immunoperoxidase staining for NF showing positive tumor cells, original magnification ×400) and neuroendocrine markers chromogranin, synaptophysin and CD56. Immunohistochemical reaction for antibodies to TTF-1 was negative.

After the surgery the patient was asymptomatic. The serum NSE and chromogranin values were within normal limits and the subsequent CT scans were normal without any evidence of remaining disease. Consequently, despite the aggressive nature of this metastatic neoplasm, the patient remained in complete remission following a multidisciplinary approach for a long period of time.

## Discussion

Merkel cell carcinoma is a rare neuroendocrine tumor of the skin, accounting for less than 1% of cutaneous malignancies. The origin of this cutaneous neuroendocrine tumor is thought to be the Merkel cells or the skin-pressure receptors (Poulsen 2004). Merkel cell carcinoma tends to grow fast and metastasize to other parts of the body. Usually it spreads to nearby lymph nodes initially and then may spread to liver, bone, lungs or brain, where it can interfere with the functioning of this organs (Tai et al. 2000a). Even under treatment, this type of carcinoma commonly metastasizes beyond skin. The treatment of this tumor should be based on a multidisciplinary approach with surgery, chemotherapy and radiotherapy (Eng et al. 2007; Ott et al. 1999; Eng et al. 2004a; Eng et al. 2004b). First of all the surgical remove of the tumor is very important and should take place when it is feasible. Additionally, chemotherapy regimens like cisplatin-etoposide (or carboplatin-etoposide, topotecan, CAV) can be used and radiotherapy can be administered in the specific involved fields (Tai et al. 2000b; Fenig et al. 1997).

Metastases to the thyroid gland are rare and may present diagnostic difficulties not only in the cytological specimens but also in the histological specimens. The most common primary tumors that metastasise to the thyroid gland are kidney cancers, colorectal cancers, lung cancers, breast cancers and sarcomas (Chung et al. 2012). In fact, metastasis from Merkel cell carcinoma to the thyroid gland is an exceptionally rare clinical condition. A rigorous search of the literature disclosed only one similar case in which the confirmation of the thyroid gland infiltration was based on the findings of fine needle aspiration FNA (Stoll et al. 2010).

Therefore, the present case is very interesting and unique because the diagnosis of the metastatic infiltration of thyroid gland from Merkel cell carcinoma was based on the histological findings on the surgical removed right thyroid lobe. During the diagnostic approach, apart from the general morphology findings in the hematoxylin-eosin-staining, the role of immunohistochemistry was very helpful. In particular, immunohistochemical stains for CK20, TTF-1 and neurofilaments are useful markers for the immunohistochemical distinction between Merkel cell carcinoma from small cell carcinoma of the lung (Leech et al. 2001; Bobos et al. 2006). More specifically, Merkel cell carcinoma is positive for the immunohistochemical antibodies to CK20 and NF and negative for TTF1 while small cell carcinoma is positive for TTF1 and negative for CK20 and NF (Bobos et al. 2006).

## Conclusions

In conclusion, the rare case of metastatic infiltration of the thyroid gland by a Merkel cell carcinoma based on histological and immunohistochemical findings was described. This case report is of clinical significance indicating that by any abnormal finding in the thyroid gland in patients with a malignant disease, the diagnostic approach should always contain consideration of metastasis from the primary tumor.
